# Fit to Perform: A Profile of Higher Education Music Students’ Physical Fitness

**DOI:** 10.3389/fpsyg.2020.00298

**Published:** 2020-03-05

**Authors:** Liliana S. Araújo, David Wasley, Emma Redding, Louise Atkins, Rosie Perkins, Jane Ginsborg, Aaron Williamon

**Affiliations:** ^1^Centre for Performance Science, Royal College of Music, London, United Kingdom; ^2^Faculty of Medicine, Imperial College London, London, United Kingdom; ^3^Trinity Laban Conservatoire of Music and Dance, London, United Kingdom; ^4^Cardiff School of Sport, Cardiff Metropolitan University, Cardiff, United Kingdom; ^5^Royal Northern College of Music, Manchester, United Kingdom

**Keywords:** cardiovascular fitness, fitness screening, flexibility, health-related fitness, music, performance, physical activity, strength

## Abstract

The physical demands of music making are well acknowledged, but understanding of musicians’ physical and fitness profiles is nonetheless limited, especially those of advanced music students who are training to enter music’s competitive professional landscape. To gain insight into how physical fitness is associated with music making, this study investigated music students’ fitness levels on several standardized indicators. Four hundred and eighty three students took part in a fitness screening protocol that included measurements of lung function, flexibility (hypermobility, shoulder range of motion, sit and reach), strength and endurance (hand grip, plank, press-up), and sub-maximal cardiovascular fitness (3-min step test), as well as self-reported physical activity (IPAQ-SF). Participants scored within age-appropriate ranges on lung function, shoulder range of motion, grip strength, and cardiovascular fitness. Their results for the plank, press-up, and sit and reach were poor by comparison. Reported difficulty (22%) and pain (17%) in internal rotation of the right shoulder were also found. Differences between instrument groups and levels of study were observed on some measures. In particular, brass players showed greater lung function and grip strength compared with other groups, and postgraduate students on the whole were able to maintain the plank for longer but also demonstrated higher hypermobility and lower lung function and cardiovascular fitness than undergraduate students. Seventy-nine percent of participants exceeded the minimum recommended weekly amount of physical activity, but this was mostly based on walking activities. Singers were the most physically active group, and keyboard players, composers, and conductors were the least active. IPAQ-SF scores correlated positively with lung function, sit and reach, press-up and cardiovascular fitness suggesting that, in the absence of time and resources to carry out comprehensive physical assessments, this one measure alone can provide useful insight into musicians’ fitness. The findings show moderate levels of general health-related fitness, and we discuss whether *moderate* fitness is enough for people undertaking physically and mentally demanding music making. We argue that musicians could benefit from strengthening their supportive musculature and enhancing their awareness of strength imbalances.

## INTRODUCTION

Behind the aesthetic and musical qualities of performance, music making can be a physically demanding activity that involves high levels of energy expenditure and elevated cardiovascular activity, often associated with augmented levels of psychosocial stress and anxiety (Fredrikson and Gunnarsson, 1992; Yoshie et al., 2009; Baadjou et al., 2011; Clark et al., 2011; Wells et al., 2012; Williamon et al., 2013; Studer et al., 2014; Vellers et al., 2015). Variations in physiological signs of stress, energy expenditure, and cardiac demands have been documented and related to musicians’ physical characteristics, instrument type, and the tempo of music performed (Iñesta et al., 2008; Williamon et al., 2013; Vellers et al., 2015; Romero et al., 2016), suggesting that the physical demands of performance are multiple and changeable. Consequently, musicians—who are sometimes referred to as ‘athletes of the upper body’ (Quarrier, 1993; Baadjou et al., 2015)—could benefit from being physically and mentally fit in order to perform at the highest levels.

One would expect musicians to show excellent upper body fitness. As an example, rock drumming has been suggested as an alternative to more traditional forms of physical activity due to its high energy demands, equivalent to moderate or vigorous activity (De La Rue et al., 2013; Romero et al., 2016). However, little is known about the fitness levels or indeed the physical characteristics required of musicians to meet these physical demands. Conversely, the existing evidence reveals a high incidence of playing-related musculoskeletal disorders (PRMDs) (Joubrel et al., 2001; Wu, 2007; Brusky, 2010; Ackermann et al., 2012; Moraes and Antunes, 2012; Kok et al., 2013) and pain in the upper body (Engquist et al., 2004; Cruder et al., 2017), as well as pressure to perform and performance anxiety among musicians from early ages (Wesner et al., 1990; van Kemenade et al., 1995; Kenny et al., 2004; Kenny and Ackermann, 2015; Gembris et al., 2018). Research has identified numerous risk factors associated with reported PMRDs and pain, such as playing posture (Nyman et al., 2007; Cruder et al., 2017), hypermobile joints (Dawson, 2002), extended time playing instruments in constrained working conditions (Leaver et al., 2011), and performance anxiety (Kenny and Ackermann, 2015). Previous studies have also suggested that musicians’ health-promoting behaviors, including engagement with physical activity, are limited (Kreutz et al., 2008, 2009; Nawrocka et al., 2013; Panebianco-Warrens et al., 2015; Araújo et al., 2017). A lack of physical activity, especially when combined with stressful working environments that encourage long periods of practice and competition, can lead to negative health consequences including locomotion and musculoskeletal problems (Ackermann and Adams, 2004; Wu, 2007; Rickert et al., 2014). Thus, the evidence contributes to a somewhat paradoxical picture where musicians’ alleged ‘athletic’ prowess contrasts markedly with their experiences of ill-health.

To understand how musicians engage physically with music making and the potential impact on their health and wellbeing, it is pertinent to know more about their physical readiness to perform. Of the studies exploring the physical characteristics of musicians, Driscoll and Ackermann (2012) have provided the most comprehensive anthropometric and musculoskeletal screening protocol for professional orchestral musicians, covering range of movement, dexterity, and strength. Their findings show that, as expected, men had better strength overall than women, upper string players (i.e., violin and viola) had the widest range of motion, brass players had the highest grip strength while string players had the lowest, brass players had the longest forearms, and 8.2% of participants met the criteria for possible joint laxity and hypermobility (using a Beighton cut-off ≥ 5). While this study’s relevance in providing anthropomorphic and range of motion estimates is undisputed, further information is needed on how musicians compare with published norms on standardized measures. Also, there is currently a lack of insight into the physical and fitness characteristics of advanced music students, those in higher education who are in the midst of intensive training to enter a demanding music profession mostly characterized by a portfolio of self-managed roles in a gig economy (Bennett, 2016; Gross et al., 2018).

Another source of data pertaining to musicians’ physical fitness can be found in studies examining the impact of physical activity and exercise on reactivity to psychosocial stress (Wasley et al., 2012) or for rehabilitation purposes (Chan et al., 2000, 2013b, 2014; Ackermann et al., 2002; Kava et al., 2010; Andersen et al., 2017). For example, in a study examining the impact of an exercise program on stress reactivity with 46 conservatoire music students (mean age = 21 years), Wasley et al. (2012) reported healthy body mass index (BMI) and average aerobic fitness (VO_2__max_) and found that fitter individuals experienced lower anxiety after performing. Chan et al. (2013a, 2014) designed an exercise program focused on strengthening supporting musculature in the neck, shoulder, abdomen, spine, and hips. Their findings showed a positive impact of exercise on reducing self-reported PRMDs and ratings of perceived exertion. Chan et al. (2013b) also investigated the effects of a video-recorded exercise program and found that orchestral musicians perceived a positive impact on strengthening muscles, increasing ease of movement and improving flexibility. With undergraduate music students, Ackermann et al. (2002) examined the effect of a strengthening and endurance exercise program on physical and self-reported fitness measures. These included isokinetic and isometric measures using dynamometer data in two planes of action (horizontal and vertical), records of weights and range of motion in each exercise, as well as intensity and frequency of PRMDs and perceived exertion. The results revealed significant increases in dynamometer measures only in the horizontal plane of motion and improvements in the number of repetitions with increased weight. They also showed a positive effect on perceived exertion during performance and daily living tasks but no significant impact on decreasing perceived intensity and frequency of PRMDs. Kava et al. (2010) investigated the effects of trunk endurance exercises on instrumental performance with 14 university music students. Results showed increases in trunk muscle stamina and decreases in perceived level of pain, fatigue, and level of exertion while playing. Finally, a study by Andersen et al. (2017) investigated the impact of specific strength training and general fitness training on instrumental playing among orchestral musicians. A parallel randomized control design was employed with 23 musicians allocated to the two interventions, each consisting of 20 mins of supervised exercise three times per week for 9 weeks. Results showed that both interventions had a positive impact on self-assessed instrumental playing, and overall, musicians were satisfied with each training approach. They reported feeling stronger, especially after general fitness training. There was a significant reduction in pain intensity after the strength training and a significant increase in aerobic capacity after the general fitness training.

Together, these studies show a positive impact of increased physical activity and instrument-specific exercise training on reducing perceptions of pain, fatigue, and anxiety, as well as perceived increases in strength and flexibility. However, in most cases, baseline information on levels of fitness based on published norms was not reported, restricting our understanding of musicians’ physical and fitness characteristics overall. Given that musicians’ readiness to meet the physical demands of making music is in question, while only limited evidence is available, this article describes an investigation of advanced music students’ physical characteristics and fitness levels in comparison with norms on standardized fitness indicators. We report differences between specific instrument groups and at different levels of musical training. In doing so, we hope to highlight areas of fitness that require further investigation and possible intervention, informing the development of effective and appropriate exercise training programs for musicians.

## Materials and Methods

This study arises from *Musical Impact*, an interdisciplinary project investigating the health and wellbeing of musicians studying and working in Europe. The project has three core strands: (1) Fit to Perform explores the attitudes, perceptions, and behaviors of musicians toward health and wellbeing, as well as their experience of chronic and acute health problems and their general fitness for performance; (2) Making Music investigates the physical and mental demands faced by musicians as they practice and perform; and (3) Better Practice examines strategies for effective health education in music conservatoires. This article focuses on Fit to Perform and, specifically, on a selection of health-related fitness measurements taken in Stage 3 of the protocol (see Procedure) to investigate physical characteristics and fitness indicators among higher education music students, as well as their levels of engagement in weekly physical activity.

### Participants

Four hundred and eighty three musicians (286 women, 197 men) studying in higher education were recruited in person and by email from ten conservatoires, nine from the UK and one from southern Switzerland, over a period of 9 years (2006–15). Sample characteristics, including nationalities, performance specialisms and genres, and institutions of study, are reported in full by Araújo et al. (2017); for ease of comparison with new data on physical characteristics and fitness indicators, they are summarized here in Table 1. Ninety-five percent of musicians who volunteered for the study identified themselves as specializing in Western classical music, which reflects the nature of conservatoire training at the participating institutions. The mean height of the sample (*N* = 483) was 1.70 m (SD ± 0.09, range 1.49–1.97), 1.65 m ± 0.06 for women and 1.77 m ± 0.07 for men. The mean weight was 64.77 kg (±11.20, range 42–112), and BMI was 22.38 kg/m^2^ (±2.90, range 16.69–32.95). Women’s mean weight was 60.03 kg (±8.32) and BMI 22.12 kg/m^2^ (±2.69), while men’s mean weight was 71.66 kg (±11.28) and BMI 22.75 kg/m^2^ (±3.15), which are normal values for both groups. The average systolic blood pressure (*n* = 205) was 115.82 mmHG (±12.74, range 92.67–156.00), diastolic was 68.97 mmHg (±8.27, range 51.00–96.00), and mean resting heart rate was 69.92 bpm (±10.79, range 43.00–104.67), showing resting blood pressure within the normal range.

**TABLE 1:** The number of women and men according to instrument group, primary performance genre, and year and institution of study (Araújo et al., 2017), followed by means and standard deviations (M ± SD) of body composition and cardiovascular data.

	**Women *n* = 286 (59%)**	**Men *n* = 197 (41%)**	**Totals *N* = 483**	**%**
**Instrument group**				
Strings	110	64	174	36%
Keyboard	51	45	96	20%
Woodwinds	66	27	93	19%
Brass	12	28	40	8%
Voice	38	11	49	10%
Percussion	6	8	14	3%
Other	3	14	17	4%
				100%
**Performance genre**				
Classical	267	190	457	95%
Non-classical (pop, jazz, folk)	19	7	26	5%
				100%
**Year of study**				
Undergraduate (UG) year 1	131	102	233	48%
UG year 2	14	19	33	7%
UG year 3	15	16	31	6%
UG year 4	15	10	25	5%
Postgraduate (PG) year 1	77	33	110	23%
PG year 2	26	13	39	8%
PG other	8	4	12	3%
				100%
**Institution of study**				
Birmingham Conservatoire (United Kingdom)	10	4	14	3.0%
Conservatorio della Svizzera Italiana (CH)	35	31	66	13.7%
Guildhall School of Music and Drama (United Kingdom)	4	0	4	0.8%
Leeds College of Music (United Kingdom)	2	3	5	1%
Royal Central School of Speech and Drama (United Kingdom)	17	2	19	3.9%
Royal College of Music (United Kingdom)	149	114	263	54.5%
Royal Conservatoire of Scotland (United Kingdom)	10	6	16	2.9%
Royal Northern College of Music (United Kingdom)	49	31	80	16.6%
Royal Welsh College of Music and Drama (United Kingdom)	6	4	10	2.1%
Trinity Laban Conservatoire of Music and Dance (United Kingdom)	4	2	6	1.2%
				100%
		
**Body composition**		**M ± SD**		
		
Height (m)	1.65 ± 0.06	1.77 ± 0.07	1.70 ± 0.09	
Weight (kg)	60.03 ± 8.32	71.66 ± 11.28	64.77 ± 11.20	
BMI (kg/m^2^)	22.12 ± 2.69	22.75 ± 3.15	22.38 ± 2.90	
				–
		
**Cardiovascular data**		**M ± SD**		
		
Systolic blood pressure (mmHg)	111.49 ± 11.27	122.89 ± 11.86	115.82 ± 12.74	
Diastolic blood pressure (mmHg)	68.11 ± 7.67	70.36 ± 9.04	68.97 ± 8.27	
Resting heart rate (bpm)	71.88 ± 9.77	66.72 ± 11.65	69.92 ± 10.79	
				–

**Strings:* violin, viola, viola de Gamba, cello, double bass, guitar (classical and electric), and harp; *Keyboard:* accordion, piano, organ, harpsichord, and historical keyboards; *Woodwinds:* flute, recorder, clarinet, oboe, bassoon, and saxophone; *Brass:* cornet, euphonium, horn, trombone, trumpet, and tuba; *Other:* composition and conducting.*

### Procedure

**FIGURE 1:**
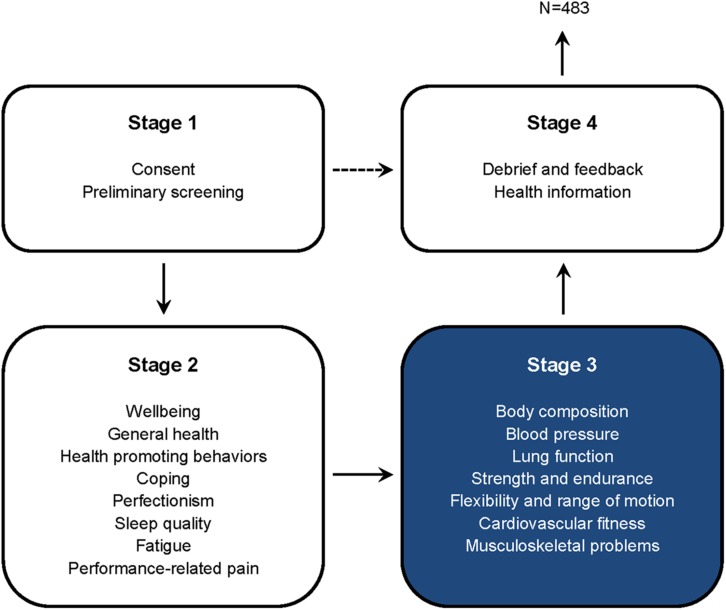
Flow of participants involved in the Fit to Perform screening protocol. This article focuses on a selection of measures from Stage 3 (*N* = 483), an assessment of music students’ physical and fitness profiles. 32 of 515 prospective participants were excluded from analyses.

The Fit to Perform screening protocol was developed as a physical and mental health assessment package for musicians, first compiled in 2006 and then expanded and refined in 2013. Assessments were conducted with individual musicians and consisted of four stages; this article reports the results of a selection of measurements from Stage 3, focusing on health-related fitness indicators. The development of the protocol and all component measures (per stage) are described by Araújo et al. (2017) and shown here in Figure 1.

Prior to participation, musicians were sent an information sheet that included instructions on alcohol, caffeine, and food intake prior to the assessment (Hoffman, 2006). Each assessment was allocated 90 min in total and was facilitated by at least three members of the research team trained to follow the detailed protocol consistently when administering the set measures. Assessments took place at each of the participating conservatoires at a pre-arranged date and time. Ethical approval for the research was granted by an independent sub-committee of the Conservatoires UK Research Ethics Committee.

### Stage 3 Measures

Stage 3 of the Fit to Perform screening protocol lasted 30–35 min and included measures of body composition, resting blood pressure, lung function, strength and endurance, flexibility, and cardiovascular capacity (Tsigilis et al., 2002; Vanhees et al., 2005; Hoffman, 2006; ACSM, 2014). A list of measures and their abbreviations are provided in Table 2.

**TABLE 2:** Measures, abbreviations, and units used in the Fit to Perform screening protocol.

**Measure**	**Abbreviation**	**Units**
**Lung function**		
Forced expiratory volume	FEV_1_	Liters
Forced expiratory volume of predicted value	FEV_1_%pred	Percentage
Forced vital capacity	FVC	Liters
Forced vital capacity of predicted value	FVC%pred	Percentage
FEV_1_/FVC ratio	FEV_1_/FVC%	Percentage
FEV_1_/FVC ratio of predicted value	FEV_1_/FVC%pred	Percentage
**Flexibility and range of motion**		
Apley’s test 1 right	AT 1_R	Percentage of Yes counts
Apley’s test 1 right with reported pain	AT 1_R pain	Percentage of Yes counts
Apley’s test 1 left	AT 1_L	Percentage of Yes counts
Apley’s test 1 left with reported pain	AT 1_L pain	Percentage of Yes counts
Apley’s test 2 right	AT 2_R	Percentage of Yes counts
Apley’s test 2 right with reported pain	AT 2_R pain	Percentage of Yes counts
Apley’s test 2 left	AT 2_L	Percentage of Yes counts
Apley’s test 2 left with reported pain	AT 2_L pain	Percentage of Yes counts
Beighton score	Beighton	Score
Stretch test with right arm	R stretch	Score
Stretch test with left arm	L stretch	Score
Sit and reach	Sit and reach	Centimeters (cm)
**Strength and endurance**		
Hand grip – right	HG-R	Kilograms (kg)
Hand grip – left	HG-L	Kilograms (kg)
Plank	Plank	Seconds
Press-up	Press-up	Number of press-ups
**Cardiovascular fitness**		
YMCA 3-min step test: Recovery heart rate	RecHR	Beats per minute (bpm)
**Physical activity**	IPAQ-SF	
Walking	Walking	METmin/week
Moderate	Moderate	METmin/week
Vigorous	Vigorous	METmin/week
Total physical activity	Total PA	METmin/week

#### Blood Pressure

Resting blood pressure was measured on the right arm while the participant was sitting, using an Omron M2 monitor (Indonesia). Three readings were taken, and the mean was calculated.

#### Height and Weight

Bare foot height (m) (Seca 213, Germany) and weight (kg) (Seca 803, Germany) were obtained from which body mass index (BMI) was derived using the standard calculation (kg/m^2^).

#### Lung Function

Lung function was measured using a Micro 1 (Carefusion, United Kingdom) spirometer to obtain forced expiratory volume (FEV_1_), forced vital capacity (FVC), and the FEV_1_/FVC ratio, as well as predicted values for each parameter. The best of three good attempts was recorded.

#### Shoulder Range of Motion

Shoulder range of motion was assessed using the Apley scratch test (Woodward and Best, 2000; Ackermann and Driscoll, 2010). The test consists of two tasks performed with each arm at a time; Apley’s test 1 consists of putting the hand behind the head (abduction and external rotation) first with the right and then with the left arm. Apley’s test 2 consists of putting the hand up behind the back (abduction and internal rotation) first with the right and then with the left arm. The ability to complete the task (i.e., yes or no) with right and left hands, as well as reports of pain while doing each task, were noted.

#### Hypermobility

Hypermobility was assessed using the Beighton hypermobility score following the instructions by Beighton et al. (2011), as also recommended by the Hypermobility Syndromes Association^1^ and the United Kingdom’s National Health Service (NHS). Scores range from 0 (no points in any of the nine joints assessed) to 9 (laxity reported in all nine joints), with higher scores indicating higher laxity and generalized hypermobility. A score of 4 in 9 symptoms is usually considered as identifying joint laxity problems (Beighton et al., 2011).

#### Shoulder Flexibility

Shoulder flexibility was measured using the shoulder reach/stretch test (adapted from FitnessGram^®^ by The Cooper Institute)^2^ on both the right and left sides. A scoring system of four points was used as an alternative to the yes/no score, with 1 poor (fingertips > 5 cm apart), 2 fair (fingertips not touching but <5 cm apart), 3 good (fingers touching), and 4 excellent (fingers overlap). When participants could not reach the back with one or both hands, a score of 0 (zero) was given.

#### Sit and Reach

Flexibility of lower back and hamstring were assessed based on Hoffman’s protocol (Hoffman, 2006) using a standard sit and reach box (zero point at 23 cm). The best score out of three attempts was recorded.

#### Hand Grip Strength

Grip strength was assessed using a hand dynamometer (Camry Digital Hand Dynamometer, Model EH101, China). Following the protocol by Hoffman (2006) and Ackermann and Driscoll (2010), participants held the dynamometer with the elbow at 90° and squeezed it as hard as they could for a few seconds. Mean grip strength for each hand was calculated across three attempts.

#### Plank

Core strength and endurance was assessed through a held forearm plank prone position for up to 60 s (Strand et al., 2014). Time to fatigue or success in completing the task within 60 s were noted.

#### Press-Up

Upper body and core strength and endurance were measured by counting the number of press-ups performed correctly within 60 s (modified version for women) (Hoffman, 2006). The total number of completed press-ups was noted.

#### Cardiovascular Fitness

Sub-maximal cardiovascular fitness was assessed using the YMCA 3-min step test (30 cm standard step box). Recovery heart rate (RecHR; bpm) was taken at 1 min post exercise (Hoffman, 2006; Morrow et al., 2015) using a Polar S610 (Finland) heart rate monitor. Using RecHR, results were placed within one of seven categories, ranging from 1 excellent to 7 very poor, adjusted for age for both women and men.

#### Physical Activity

In order to explore associations between objective fitness levels and self-reported engagement in physical activity, the International Physical Activity Questionnaire Short-Form (IPAQ-SF)^3^ was administered. The IPAQ-SF has been used extensively and is recommended for monitoring and longitudinal studies. Reports of time spent walking and doing vigorous and moderate intensity activity in the last 7 days were collected. Time and days doing physical activity were converted to Metabolic Equivalents (MET) per min per week resulting in a continuous score used for purposes of analysis. The following MET values were used as recommended by the IPAQ scoring protocol: walking = 3.3 METs, moderate physical activity = 4.0 METs, vigorous physical activity = 8.0 METs. It is suggested that a range between 500 and 1000 MET-minutes per week is necessary to achieve health benefits, which is equivalent to spending 5 or more days in any combination of walking, moderate-intensity or vigorous intensity activities, or 5 or more days doing at least 30 min per day of a combination of walking and moderate intensity activities (Office of Disease Prevention and Health Promotion, 2008).

### Data Treatment and Analyses

Using a cross-sectional and correlational design, data from female and male music students of different instrument groups and levels of study were analyzed using SPSS (v. 24). On the basis of screening to take part and after data preparation, 32 of 515 prospective participants were excluded from analyses, resulting in a final sample of 483 participants (see Araújo et al., 2017).

The normality of the distribution was explored using Kolmogorov–Smirnoff tests and analysis of histograms, which showed that most of the variables were not normally distributed. Homogeneity of variance across groups (sex, instrument group, and academic level) was also not verified. Subsequent analyses were therefore performed using non-parametric tests. Analyses were undertaken examining differences in physical characteristics and fitness measures based on sex, instrument group, and level of study using Mann–Whitney *U* and Kruskal–Wallis tests with appropriate pairwise comparisons and corrections. Wilcoxon signed rank tests were used to compare within-subject differences on two related tasks (e.g., between measurements taken on the right and left sides). Effect sizes were estimated using $r=\frac{z}{\sqrt{N}}$ (Field, 2013), and the alpha level was set at 5%. Associations between self-reported physical activity (IPAQ-SF) and the other health-related fitness indicators used in the Fit to Perform screening protocol were explored through partial non-parametric correlational analyses (Spearman’s rho), controlling for sex due to the observed differences between men and women. Where appropriate, sample size reported varies from 483 due to part of the sample (*n* = 205) completing a shortened version of the protocol.

## Results

The results are presented in two parts. The first describes the physical fitness levels of our sample of higher education music students, reporting data for the entire sample including comparisons with published norms and differences between women and men. Where observed, differences between instrument groups and levels of study are also reported. Analyses of differences by sex within instrument groups were not performed due to the unavoidable differences between the numbers of men and women in each group (see Supplementary Table S1 for descriptive statistics by sex and instrument group). There was also an uneven sex distribution between undergraduate and postgraduate students, so separate Mann–Whitney tests were run when relevant for further clarification of results (see Supplementary Table S2 for descriptive statistics by sex and level of study). In the second part, correlations between self-reported physical activity (IPAQ-SF) and the other health-related fitness indicators are presented.

### Physical Fitness Levels

Table 3 shows descriptive statistics for each measure used in the screening protocol for the entire sample and divided by sex. Sex differences were examined using Mann–Whitney *U* tests. Comparisons with normative values (where available) are addressed in the following sections for each variable.

**TABLE 3:** Descriptive statistics for the health-related fitness indicators used in the Fit to Perform screening protocol by sex, as well as Mann–Whitney *U* tests for differences by sex.

**Measure**	**Women**	**Men**	**Total**	**U, p**
**Lung function**		**M ± SD**		
		
FEV_1_	2.890.56	3.940.94	3.290.88	**1577.50, *p* < 0.001**
FEV_1_%pred	89.5315.66	90.3118.61	89.8216.80	4736.00, *p* = 0.599
FVC	3.080.63	4.441.24	3.601.11	**1401.50, *p* < 0.001**
FVC%pred	83.0115.65	85.6821.07	84.0217.90	4676.50, *p* = 0.502
FEV_1_/FVC%	96.414.62	93.018.21	95.126.43	**4020.00, *p* = 0.021**
FEV_1_/FVC%pred	114.395.61	112.5810.04	113.707.63	4700.50, *p* = 0.538
		
**Flexibility and range of motion**		**% (n)**		
		
AT 1_R	96% (122)	97% (76)	97% (198)	4885.00, *p* = 0.600
AT 1_R pain	4% (5)	6% (5)	5% (10)	4830.50, *p* = 0.426
AT 1_L	97% (123)	94% (73)	96% (196)	4791.50, *p* = 0.270
AT 1_L pain	2% (2)	5% (4)	3% (6)	4777.00, *p* = 0.144
AT 2_R	81% (103)	73% (57)	78% (160)	4555.50, *p* = 0.179
AT 2_R pain	15% (19)	19% (15)	17% (34)	4741.50, *p* = 0.426
AT 2_L	91% (116)	89% (69)	90% (185)	4810.50, *p* = 0.501
AT 2_L pain	5% (6)	9% (7)	6% (13)	4742.50, *p* = 0.227
		
		**M ± SD**		
		
Beighton	2.221.95	1.551.70	1.971.88	**3952.50, *p* = 0.013**
R stretch	3.610.88	3.531.14	3.580.99	4811.00, *p* = 0.634
L stretch	2.971.39	2.881.46	2.941.41	4843.00, *p* = 0.769
Sit and reach	29.4110.39	23.9411.60	27.3311.16	**3616.50, *p* = 0.001**
		
**Strength and endurance**		**M ± SD**		
		
HG-R	26.694.82	39.387.46	31.868.69	**4066.50, *p* < 0.001**
HG-L	25.484.54	37.526.57	30.398.06	**3869.50, *p* < 0.001**
Plank	51.7613.72	55.2213.15	53.0713.58	4739.00, *p* = 0.585
Press-up	10.878.47	20.5013.38	14.5411.57	**2664.50, *p* < 0.001**
		
**Cardiovascular fitness**		**M ± SD**		
		
RecHR	105.5716.92	99.2317.65	102.9817.48	**22249.50, *p* < 0.001**
		
**Physical activity**		**M ± SD**		
		
Walking	1382.101169.37	1001.42828.27	1237.261066.70	4156.00, *p* = 0.053
Moderate	503.94815.38	503.85713.24	503.90776.30	4882.00, *p* = 0.859
Vigorous	604.721051.14	1130.261765.53	804.681387.23	**3906.50, *p* = 0.008**
Total PA	2490.762002.48	2635.532317.65	2545.842123.48	4881.50, *p* = 0.862

*M, mean; SD, standard deviation. Measures, abbreviations, and units for each measure are provided in Table 2. Highlighted values in bold show statistically significant results.*

#### Lung Function

Participants showed normal values in lung capacity (Barreiro and Perillo, 2004), with only 1% of participants (*n* = 4) achieving FEV_1_/FVC% values below 80%, the cut-off point for limited lung function. As expected, differences were observed between women and men: men had higher FEV_1_ (*U* = 1577.50, *p* < 0.001, *r* = 0.57) and FVC (*U* = 1401.50, *p* < 0.001, *r* = 0.60) with medium effect sizes for the differences but a lower FEV_1_/FVC ratio (*U* = 4020.00, *p* = 0.021, *r* = 0.16), with a low effect size.

Differences between instrument groups and levels of study were observed. Brass players showed higher values than other instrument groups on FEV_1_ (keyboard < brass, *H* = −62.54, *p* < 0.005, *r* = 0.26; strings < brass, *H* = −54.63, *p* = 0.005, *r* = 0.26; voice < brass, *H* = 60.60, *p* < 0.010, *r* = 0.25; woodwinds < brass, *H* = −52.60, *p* = 0.017, *r* = 0.23); FEV_1__%_pred (strings < brass, *H* = −49.81, *p* = 0.016, *r* = 0.23); FVC (strings < brass, *H* = −56.58, *p* < 0.005, *r* = 0.27; voice < brass, *H* = 62.79, *p* < 0.005, *r* = 0.26; keyboard < brass, *H* = −60.587, *p* < 0.010, *r* = 0.25); and FVC%pred (strings < brass, *H* = −47.95, *p* = 0.025, *r* = 0.23) (see Table 4). Undergraduate students displayed higher FEV_1_ (*U* = 4150.00, *p* = 0.011, *r* = 0.17) than postgraduate students (see Table 5). Further separate analysis for women and men showed significant differences only between undergraduate and postgraduate men (*U* = 537.50, *p* = 0.034, *r* = −0.24) but not women. No differences were observed for FEV_1_ with predicted values based on sex, age and height which suggests, along with the small effect sizes observed, low practical importance.

**TABLE 4:** Descriptive statistics for the health-related fitness indicators used in the Fit to Perform screening protocol by instrument group, as well as Kruskal–Wallis tests for differences between groups.

**Measure**	**Strings**	**Keyboard**	**Woodwinds**	**Brass**	**Voice**	**Percussion**	**Other**	**H, p**
**Lung function**				**M ± SD**				
		
FEV_1_	3.200.92	3.100.96	3.210.67	4.110.95	3.050.53	3.720.95	4.130.97	**22.75, *p* = 0.001**
FEV_1_%pred	87.4618.64	87.5320.50	90.2313.86	100.8111.40	88.7212.98	82.0022.07	98.8011.14	**14.60, *p* = 0.024**
FVC	3.411.03	3.311.04	3.761.32	4.511.15	3.250.65	4.340.80	4.421.09	**24.56, *p* < 0.001**
FVC%pred	79.6017.60	80.4118.42	90.4320.09	93.2413.79	81.7514.14	80.0016.64	89.2010.31	**16.71, *p* = 0.010**
FEV_1_/FVC%	96.374.87	96.475.19	92.828.76	93.107.13	96.444.17	87.6712.06	94.206.26	12.26, *p* = 0.056
FEV_1_/FVC%pred	115.285.82	115.256.06	110.5510.21	111.868.65	114.975.29	106.3314.57	114.207.79	12.07, *p* = 0.060
		
**Flexibility and range of motion**				**% (n)**				
		
AT 1_R	96% (65)	94% (30)	96% (42)	100% (21)	100% (32)	100% (3)	100% (5)	3.30, *p* = 0.771
AT 1_R pain	4% (3)	3% (1)	7% (3)	5% (1)	3% (1)	0% (0)	20% (1)	3.41, *p* = 0.755
AT 1_L	94% (64)	97% (31)	91% (40)	100% (21)	100% (32)	100% (3)	100% (5)	5.57, *p* = 0.473
AT 1_L pain	4% (3)	3% (1)	2% (1)	0% (0)	3% (1)	0% (0)	0% (0)	1.47, *p* = 0.961
AT 2_R	74% (50)	75% (24)	80% (35)	95% (20)	75% (24)	100% (3)	80% (4)	5.66, *p* = 0.462
AT 2_R pain	15% (10)	19% (6)	21% (9)	14% (3)	16% (5)	0% (0)	20% (1)	1.49, *p* = 0.960
AT 2_L	87% (59)	88% (28)	89% (39)	100% (21)	97% (31)	100% (3)	80% (4)	6.10, *p* = 0.412
AT 2_L pain	7% (5)	6% (2)	7% (3)	0% (0)	6% (2)	0% (0)	20% (1)	3.31, *p* = 0.769
		
				**M ± SD**				
		
Beighton	1.871.49	1.811.94	2.002.16	1.861.68	2.222.03	0.330.58	3.803.35	5.81, *p* = 0.445
R stretch	3.531.11	3.501.05	3.481.00	3.760.70	3.810.59	4.000.00^[1]^	3.001.73	6.83, *p* = 0.337
L stretch	3.011.42	2.591.66	2.611.48	3.480.93	3.221.16	4.000.00^[1]^	2.201.48	11.97, *p* = 0.063
Sit and reach	27.0110.53	24.9512.21	26.8410.55	24.9012.94	32.6310.42	25.338.74	28.6010.92	11.13, *p* = 0.084
		
**Strength and endurance**				**M ± SD**				
		
HG-R	31.248.74	31.147.55	31.108.63	38.139.78	29.076.23	35.3610.99	36.927.62	**31.11, *p* < 0.001**
HG-L	29.918.20	29.516.99	29.598.23	35.988.08	28.085.77	33.2810.53	35.877.34	**32.56, *p* < 0.001**
Plank	50.8516.56	53.7212.11	52.5511.59	53.5213.54	56.4411.63	60.000.00^[1]^	56.205.76	**30.88, *p* < 0.001**
Press-up	16.0713.35	13.5611.16	13.3011.92	13.627.91	14.698.74	14.3310.02	13.8017.92	2.61, p = 0.856
		
**Cardiovascular fitness**				**M ± SD**				
		
RecHR	104.4117.36	102.1616.26	101.1717.79	103.3217.79	105.1418.72	94.0019.20	103.2917.56	4.80, *p* = 0.569
		
**Physical activity**				**M ± SD**				
		
Walking	1212.261106.20	884.81768.95	1394.631280.94	1019.86747.26	1557.19988.08	2194.501709.24	739.20390.88	12.04, *p* = 0.061
Moderate	327.94563.51	380.00607.20	597.27780.59	422.86752.02	932.501151.08	560.00969.95	432.00429.33	**18.14, *p* = 0.006**
Vigorous	773.531354.43	546.251342.30	841.821273.41	560.00815.45	1262.501919.97	853.331211.50	624.00891.56	6.05, *p* = 0.417
Total PA	23133.741960.04	1811.061831.16	2833.721919.07	2002.711061.08	3752.192889.31	3607.833871.30	1795.201154.22	**18.28, *p* = 0.006**

*M, mean; SD, standard deviation. *Measures, abbreviations, and units for* each measure are provided in Table 2. *Strings:* violin, viola, viola de Gamba, cello, double bass, guitar (classical and electric), and harp; *Keyboard:* accordion, piano, organ, harpsichord, and historical keyboards; *Woodwinds:* flute, recorder, clarinet, oboe, bassoon, and saxophone; *Brass:* cornet, euphonium, horn, trombone, trumpet, and tuba; *Other:* composition and conducting. ^[*1*]^Data refer to small *n* values (*n* < 3); see Supplementary Table S1. Highlighted values in bold show statistically significant results.*

**TABLE 5:** Descriptive statistics for the health-related fitness indicators used in the Fit to Perform screening protocol by level of study, as well as Mann–Whitney *U* tests for differences by level of study.

**Measure**	**Undergraduate**	**Postgraduate**	**U, *p***
**Lung function**	**M ± SD**		
		
FEV_1_	3.440.89	3.120.85	**4150.00, *p* = 0.011**
FEV_1_%pred	92.0714.42	87.2718.91	4636.50, *p* = 0.160
FVC	3.681.01	3.501.24	4504.50, *p* = 0.086
FVC%pred	84.1814.09	83.8421.51	5119.50, *p* = 0.791
FEV_1_/FVC%	95.965.46	94.167.29	4482.50, *p* = 0.072
FEV_1_/FVC%pred	114.646.50	112.648.65	4536.50, *p* = 0.099
		
**Flexibility and range of motion**	**% (n)**		
		
AT 1_R	98% (107)	95% (91)	5055.50, *p* = 0.186
AT 1_R pain	4% (4)	6% (6)	5097.00, *p* = 0.393
AT 1_L	97% (106)	94% (90)	5049.00, *p* = 0.224
AT 1_L pain	3% (3)	3% (3)	5212.50, *p* = 0.875
AT 2_R	79% (86)	77% (74)	5137.00, *p* = 0.755
AT 2_R pain	14% (15)	20% (19)	4916.50, *p* = 0.248
AT 2_L	92% (100)	89% (85)	5064.50, *p* = 0.442
AT 2_L pain	6% (7)	6% (6)	5223.50, *p* = 0.960
		
	**M ± SD**		
		
Beighton	1.391.75	2.611.82	**3026.50, *p* < 0.001**
R stretch	3.521.12	3.640.81	5036.50, *p* = 0.523
L stretch	2.921.54	2.961.26	4966.00, *p* = 0.489
Sit and reach	26.2311.31	28.5710.92	4498.00, *p* = 0.083
		
**Strength and endurance**	**M ± SD**		
		
HG-R	32.168.52	31.288.98	23643.00, *p* = 0.115
HG-L	30.678.03	29.848.10	23900.00, *p* = 0.162
Plank	50.7114.22	30.5622.90	**2817.50, *p* < 0.000**
Press-up	13.8910.95	15.2712.25	4916.50, *p* = 0.456
		
**Cardiovascular fitness**	**M ± SD**		
		
RecHR	101.6317.49	105.7017.20	**22021.50, *p* = 0.007**
		
**Physical activity**	**M ± SD**		
		
Walking	1256.42998.42	1215.501144.23	4709.00, *p* = 0.216
Moderate	453.58615.25	561.04926.05	5150.00, *p* = 0.842
Vigorous	875.231426.66	724.581344.00	5007.50, *p* = 0.579
Total PA	2585.231901.58	2501.132359.71	4722.00, *p* = 0.229

*M, mean; SD, standard deviation. *Measures, abbreviations, and units for* each measure are provided in Table 2. Highlighted values in bold show statistically significant results.*

#### Flexibility and Range of Motion

Musicians in this sample did not generally display joint hypermobility, with only 11% of participants (*n* = 22) reporting scores above the suggested cut-off point of 4, and 5% (*n* = 10) above the cut-off point of 5 (Beighton et al., 2011). Overall, these scores are lower than previously observed in studies with musicians, where reports range up to 40% prevalence of hypermobility based on scores above the cut-off point (Larsson et al., 1993; Grahame, 2007). As expected (Beighton et al., 2011), women scored significantly higher than men (*U* = 3951.50, *p* = 0.013, *r* = −0.17).

There were no differences between instrument groups, but differences were observed between levels of study, with postgraduate students obtaining higher hypermobility scores than undergraduate students (*U* = 3026.50, *p* < 0.001, *r* = −0.37; see Table 5). Considering the tendency for women to score higher for hypermobility, these findings may reflect a sex bias as there were more women in the postgraduate group (*n* = 111 of 161 postgraduate students). Mann–Whitney tests were run separately comparing undergraduate and postgraduate women and undergraduate and postgraduate men, and both postgraduate women (*U* = 1351.50, *p* = 0.001, *r* = −0.30) and men (*U* = 334.50, *p* < 0.001, *r* = −0.21) scored significantly higher than their undergraduate peers (see Supplementary Table S2 for results by sex and level of study).

In terms of abduction and external rotation, the students on the whole showed an adequate range of motion (Apley’s test 1) as well as internal rotation up the back (Apley’s test 2) in both left and right shoulders. Reports of pain were the highest (17%) for the Apley’s test 2 on the right side. A Wilcoxon signed-rank test revealed significant differences, with low effect size, between the right and left side in Apley’s test 2 (*z* = −3.812, *p* < 0.001, *r* = 0.27). Seventy-eight percent of participants could perform internal rotation up the back with the right arm compared with 90% who could complete the task with the left arm, demonstrating range of motion imbalances between right and left sides.

The average global score on the stretch test was 3.58 (±0.99) on the right side and 2.94 (±1.41) on the left, with a significant difference observed between the two sides (z = −6.759, *p* < 0.001, *r* = 0.47). Seventy-eight percent of participants scored 4 (excellent = fingers overlapping) on the right side compared with 55% scoring 4 on the left side, which requires internal rotation of the right shoulder. Two percent (*n* = 8) could not perform the task on the right side, which requires internal rotation of the left shoulder, compared with 4% (*n* = 20) who could not perform the task on the left side, which requires internal rotation of the right shoulder. No differences were found in any of the Apley’s tests or flexibility tests between groups (sex, instrument group, or level of study).

With regards to the sit and reach test, when compared with published norms (Hoffman, 2006; ACSM, 2014), the overall score was below average, showing poor hamstring and lower back flexibility in musicians. As expected, women showed significantly greater flexibility than men (*U* = 3616.50, *p* = 0.001, *r* = −0.22). No differences were found between instrument groups or levels of study.

#### Strength and Endurance

Grip strength for women and for men met normal standards, where normative values range from 21.5–5.3 kg for women 20–24 years old and 36.8–56.6 kg for men 20–24 years old. Women’s scores were significantly lower than men’s, as expected, for both the right (*U* = 4066.50, *p* < 0.001, *r* = 0.73) and left grip (*U* = 3869.50, *p* < 0.001, *r* = 0.73). A Wilcoxon signed ranks test revealed significant differences between right and left grip strength (*z* = −10.10, *p* < 0.001) across the whole sample, with more strength in the right hand. Significant differences were observed between instrument groups on the right (*H* = 31.11, *p* < 0.001) and the left side (*H* = 32.56, *p* < 0.001) (Table 4). Pairwise comparisons showed that brass players had significantly stronger grip when compared with singers (*H* = 127.48, <0.001, *r* = 0.20), woodwinds (*H* = −107.852, *p* = 0.001, *r* = 0.19), strings (*H* = −102.73, *p* = 0.001, *r* = 0.19), and keyboard players (*H* = −97.52, *p* < 0.005, *r* = 0.17) on the right hand side. Similar results were found on left hand grip, where brass players again showed higher scores than singers (*H* = 129.59, *p* < 0.001, *r* = 0.19), woodwinds (*H* = −112.67, *p* < 0.001, *r* = 0.19), strings (*H* = −100.95, *p* = 0.001, *r* = 0.19), and keyboard players (*H* = −104.38, *p* = 0.001, *r* = 0.18). Other musicians (composers/conductors) were stronger when compared with singers on the right (*H* = −125.16, *p* = 0.030, *r* = 0.14) and the left grip (*H* = −130.79, *p* = 0.018, *r* = 0.15) and with woodwinds on the left grip (*H* = −113.88, *p* = 0.042, *r* = 0.14). No differences were observed between levels of study.

Compared with published norms, music students scored poorly overall on the plank test (Strand et al., 2014) with results below the 30th percentile for both women and men. No statistically significant differences were observed between women and men. Significant differences were observed between instrument groups (*H* = 30.88, *p* < 0.001) (Table 4) with pairwise comparisons showing that singers had significantly better results when compared with strings (*H* = −57.76, *p* < 0.001, *r* = 0.33), woodwinds (*H* = −57.21, *p* < 0.001, *r* = 0.31), and keyboard players (*H* = −46.33, *p* = 0.021, *r* = 0.23). Despite such poor scores overall, undergraduate students maintained the plank for longer than postgraduate students (*U* = 2817.50, *p* < 0.001, *r* = −0.41, Table 5). Women scored consistently low on the plank test regardless of level of study, and postgraduate men scored higher than undergraduate men (*U* = 357.00, *p* < 0.001, *r* = −0.47) (see Supplementary Table S2 for results by sex and level of study).

All participants performed their maximum number of press-ups under 60 s. Poor results were observed when compared with published norms (Hoffman, 2006), with observed differences between women and men (*U* = 2664.50, *p* < 0.001, *r* = 0.39) and both groups scoring on the whole below the 20th percentile. No differences were found between instrument groups or levels of study.

#### Cardiovascular Fitness

**FIGURE 2:**
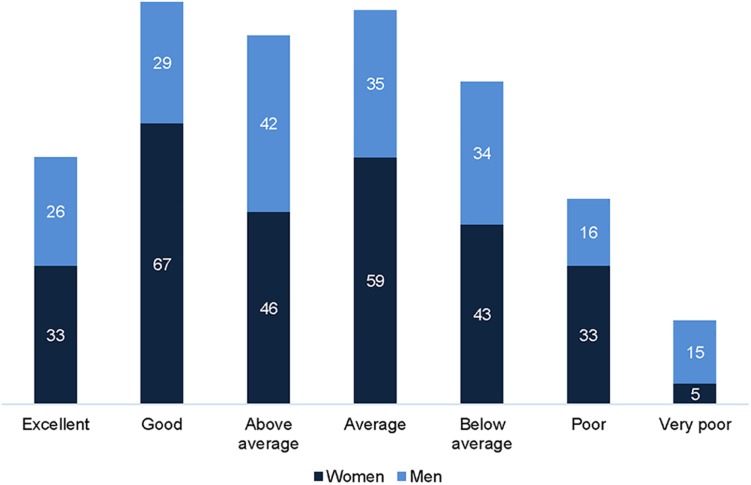
Distribution of women (*n* = 286) and men (*n* = 197) across age-adjusted heart rate recovery (RecHR) categories.

The overall mean for recovery heart rate (RecHR) was 102.98 bpm (±17.48), 105.57 bpm (±16.92) for women and 99.23 bpm (±17.65) for men, with 23% of women scoring in the category *good* and 21% of men *above average* based on mean age (Hoffman, 2006) (Figure 2). Significant differences were found on the RecHR between women and men (*U* = 22249.50, *p* < 0.001, *r* = 0.18), but no differences were found when comparing median values in the age-adjusted heart rate recovery categories.

**FIGURE 3:**
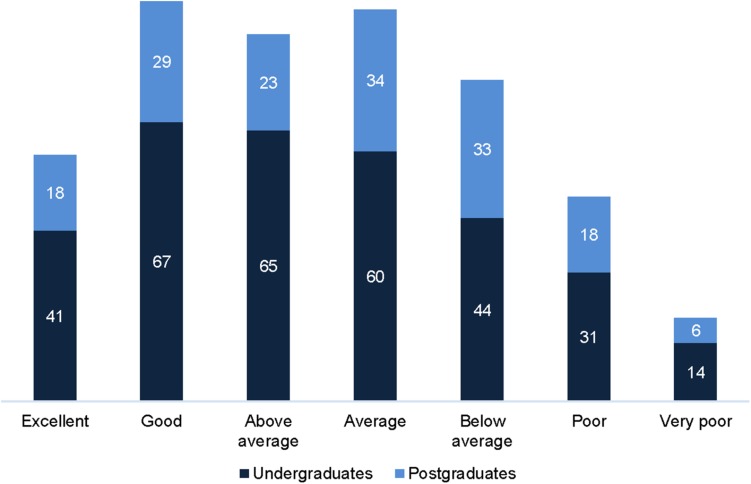
Distribution of undergraduate (*n* = 322) and postgraduate students (*n* = 161) across age-adjusted heart rate recovery (RecHR) categories.

Differences in RecHR were significant between undergraduate and postgraduate students (*U* = 22021.50, *p* = 0.007, *r* = 0.12). Undergraduate students scored mostly in the good (21%) and above average categories (20%), and postgraduate students in the average (21%) and below average (21%) categories (Figure 3). No differences were found between undergraduate and postgraduate students when analyzing women and men separately, suggesting that other factors (e.g., age, sex, or uneven distribution between groups) may have influenced the results, which is also reflected in the small effect size.

#### Physical Activity

**FIGURE 4:**
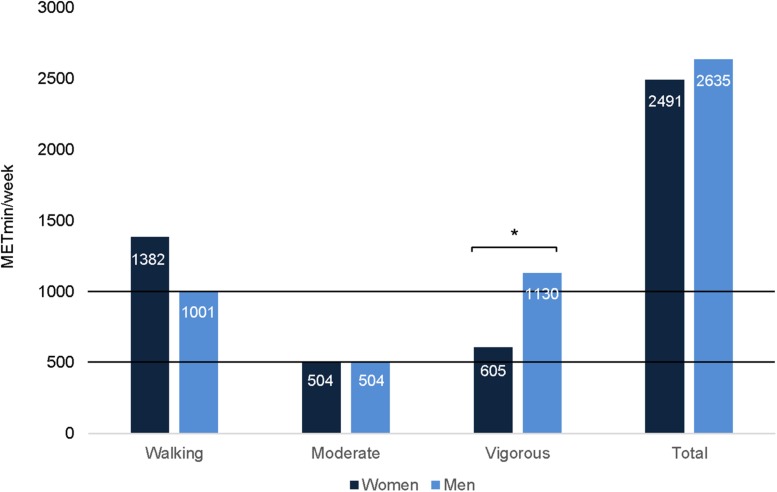
Weekly physical activity for women and men according to recommendations of 500–1000 METmin/week. Seventy-nine percent exceeded the weekly recommendations, with significant differences in vigorous activity between women and men (* *p* < 0.01).

Participants’ self-reports of physical activity indicated that 79% exceeded the recommended weekly limits of physical activity (500–1000 MET-min/week, equivalent to engaging in a combination of walking, moderate and vigorous intensity activities for 5 or more days), 10% met the recommendations, and 11% did not meet the recommendations (Office of Disease Prevention and Health Promotion, 2008; Sylvia et al., 2014; Kahlmeier et al., 2015) (Figure 4). Walking was the most frequent activity, compared with vigorous or moderate activity. If considering only moderate intensity activity, which is recommended for 150 min per week for health benefits (Kahlmeier et al., 2015), the music students were within the recommended limits, although at the lower end. Differences between women and men were observed only in vigorous activity (*U* = 3906.50, *p* = 0.008, *r* = 0.19), with men reporting a greater amount of vigorous physical activity than women, as observed in other studies (Sylvia et al., 2014).

Differences were observed between instrument groups on moderate physical activity (*H* = 18.14, *p* = 0.006, *r* = 1.27) and total physical activity (*H* = 18.28, *p* = 0.006, *r* = 1.28) (Table 4). When considering total physical activity, all groups exceeded the weekly recommendations, and significant differences were observed between groups (*p* = 0.006) but only between singers and keyboard players (*p* = 0.004, *r* = 0.26). Pairwise comparisons also showed that singers engaged significantly more in moderate physical activity than string (*p* = 0.003, *r* = 0.27) and keyboard players (*p* = 0.045, *r* = 0.21).

### Links Between Self-Reported Physical Activity and Health-Related Fitness Indicators

Partial non-parametric correlations were calculated to examine associations between self-reported physical activity and health-related fitness indicators, controlling for sex (Table 6). Results showed that self-reported physical activity was positively and significantly associated with lung function (FEV_1_, FVC, and FVC with predicted values), flexibility (sit and reach), strength and endurance (left handgrip, plank, and press-up), and cardiovascular fitness (RecHR).

**TABLE 6:** Partial non-parametric correlations between self-reported physical activity (IPAQ-SF) and the other health-related fitness indicators used in the Fit to Perform screening protocol (Spearman’s rho), controlling for sex.

**Measure**	***r*_s_, *p***
**Lung function**	
FEV_1_	**0.178, *p* = 0.011**
FEV_1_%pred	0.128, *p* = 0.068
FVC	**0.185, *p* = 0.008**
FVC%pred	**0.150, *p* = 0.032**
FEV_1_/FVC%	−0.096, *p* = 0.170
FEV_1_/FVC%pred	−0.069, *p* = 0.330
**Flexibility and range of motion**	
Beighton	−0.041, *p* = 0.565
R stretch	0.011, *p* = 0.875
L stretch	0.021, *p* = 0.762
Sit and reach	**0.216, *p* = 0.002**
**Strength and endurance**	
HG-R	0.104, *p* = 0.140
HG-L	**0.146, *p* = 0.037**
Plank	**0.310, *p* < 0.001**
Press-up	**0.288, *p* < 0.001**
**Cardiovascular fitness**	
RecHR	**−0.165, *p* = 0.019**

*The negative correlations with RecHR were, in fact, positive associations, as higher scores in RecHR indicate lower cardiovascular fitness. Highlighted values in bold show statistically significant results.*

## Discussion

The findings of this study extend the understanding of music students’ physical fitness. Existing research suggests that musicians engage relatively little in health-promoting behaviors, in particular physical activity (Kreutz et al., 2008, 2009; Panebianco-Warrens et al., 2015; Araújo et al., 2017). It is also known that physical health problems are common among musicians across almost all specialist areas and genres of performance (Zaza, 1998; Bragge et al., 2006; Ackermann et al., 2012; Steinmetz et al., 2012; Arnason et al., 2014; Kenny and Ackermann, 2015). We therefore expected our sample of higher education music students to fare poorly on standardized indicators of overall physical fitness, which was not entirely the case. Our participants showed a standard profile based on body composition characteristics (e.g., BMI), resting blood pressure, and weekly engagement in physical activity, and they scored within ranges appropriate for their age on lung function, shoulder range of motion, grip strength, and cardiovascular fitness.

While these results are generally positive in the wider context of university students’ physical profiles, it is worth considering whether this apparently healthy state is sufficient to perform music at the highest levels, especially considering the physical exertion required in the practice room and on stage, the high incidence of reported musculoskeletal problems in the upper body, and the general lack of health-promoting behaviors previously documented. Due to the physical demands of music making, we expected our sample to exceed published norms in at least upper body strength and range of motion. However, their results on the plank, press-up, and sit and reach were poor by comparison, and they reported difficulty (22%) and pain (17%) in internal rotation of the right shoulder. Some significant differences emerged between certain instrument groups and levels of study for specific measures (discussed below), raising questions about the potential impact of specialist training, skills, and selection factors on musicians’ physical fitness. It is therefore relevant to explore the specific physiological demands of making music and the role of physical fitness in relation to these demands.

In terms of lung function, our findings are in contrast with those of previous studies (Schorr-Lesnick, 1988; Deniz et al., 2006; Granell et al., 2011), which have shown that playing a wind instrument is related to decreased pulmonary function and that lung function correlates negatively with duration of practice. In fact, brass players had significantly better lung capacity than most others, including singers and woodwind players. However, if lung capacity diminishes with practice duration, as suggested in the literature, further examination is required, investigating musicians at different stages in their careers, for example, or longitudinally.

Similarly, brass players also achieved better results for both right and left grip strength compared with other musicians, with singers demonstrating the weakest grip. These represent poorer results than those found by Driscoll and Ackermann (2012), which leads us to question whether grip strength increases with years of instrument practice. However, in our analyses, no differences in grip strength were found between levels of study, leaving the impact of training on these aspects still to be examined. In addition, hand grip and upper body strength and endurance should be investigated based on the weight of the instrument and playing position. With regard to hypermobility, previous reports have suggested a high incidence of hypermobility among musicians (Larsson et al., 1993; Grahame, 2007) and potential differences between instrument groups (Larsson et al., 1993; Quarrier, 2011). The incidence rate of joint laxity in the general population is controversial and may account for up to 45% of routine rheumatology referrals (Grahame, 2008). Hypermobility is also related to age, sex, and ethnicity and tends to be higher in younger people and women (Grahame, 2008; Beighton et al., 2011). As expected, women in our study showed higher joint laxity than men, yet the incidence of hypermobility was low (5–11%), and no significant differences between instrument groups were found.

Previous research suggesting poor engagement of musicians in physical activity has mostly used general health-promoting questionnaires (e.g., Health Promoting Lifestyle Profile, Walker et al., 1987) and not specific measures of weekly physical activity or indicators of fitness levels. In our study, music students not only reported doing weekly physical activity, with satisfactory weekly levels across all activity types (Office of Disease Prevention and Health Promotion, 2008; Kahlmeier et al., 2015), but they also showed average levels of cardiovascular fitness according to age-adjusted heart rate recovery (RecHR) categories. Significant associations with amount of weekly physical activity suggest that cardiovascular fitness in music students is linked with on their engagement in physical activity and does not vary according to instrument played. In fact, partial correlations controlling for sex showed that those who engage in weekly physical activity were more flexible (sit and reach), had better results in terms of cardiovascular fitness and lung function, and had greater upper body strength and endurance as measured by the plank test and the number of press-ups completed. On the other hand, our findings suggest that these measures, in particular the sit and reach, plank, press-up, and the step test, while useful for measuring health-related fitness (Vanhees et al., 2005; ACSM, 2014), are associated with self-reported physical activity via the IPAQ-SF (Booth et al., 2003; Fogelholm et al., 2006; Hagstromer et al., 2010; Lee et al., 2011). We acknowledge the caveat that, as a self-report measure, the IPAQ may be susceptible to bias and over-rating (Hagstromer et al., 2010; Lee et al., 2011).

Additionally, while most musicians met general physical activity recommendations, 21% of the sample did not, and the majority of physical activity reported was based on walking activity which may not be sufficient to achieve full health benefits. Given the associated profile of other physical facets detailed here, and despite the significant but weak correlations with self-reported physical activity, future studies should consider monitoring and measuring levels of engagement in weekly physical activity, measured objectively, as well as implementing and evaluating specific exercise programs for musicians and their potential impact on increasing levels of physical fitness.

Previous research has shown that engagement in physical activity by university-level students is variable across sexes, subject of study, country of origin, attitudes toward health promotion, and participation in team versus individual sports (Bednarek et al., 2016). Those studying sports or physical education and participating in competitive sports achieve levels of physical activity as measured in MET-min/per week twice higher than our music students (Pastuszak et al., 2014; Fagaras et al., 2015). In this study, there was a range of physical activity undertaken across all instrument groups, but singers stood out. Previous research has suggested that singers have heightened sensitivity and attitudes toward health compared with other musicians (Schorr-Lesnick et al., 1985; Sapir et al., 1996), which may explain the higher levels of engagement in physical activity reported here. Anecdotally, most music students in our sample commented on walking or cycling to college and using gym facilities at their student accommodation to save money and stay active, which may explain the results for self-reported physical activity and cardiovascular fitness. It is, therefore, worth exploring ways of encouraging music students to sustain healthy and active lifestyles by increasing access to affordable physical activity initiatives.

Furthermore, the World Health Organization [WHO] (2010) clearly recommends muscle-strengthening activities on two or more days per week involving major muscular groups in addition to regular engagement in moderate and vigorous-intensity activity. The results emerging from the IPAQ-SF are thus limited as they do not record such muscle-strengthening activities. It has been suggested recently that the IPAQ should align better with the WHO recommendations and use tougher requirements at the moderate level of activity by, for instance, including clear criteria for what is considered ‘activity level for health’ or increasing the threshold for 1200 MET-min per week. This would be particularly important for identifying the physical activity levels of people not involved in specific physical training, thereby providing a more realistic measure of physical activity (Lee et al., 2011; Pastuszak et al., 2014).

Our findings show poor core and upper body strength and endurance (as seen in the plank and press-up results), weak lower back and hamstring flexibility (as seen in the sit and reach results) and, despite good range of motion overall, some reported difficulties in shoulder rotation in the right side. The proximal muscles involved in the plank and press-up tests have a functional relevance to supportive musculature responsible for preventing injury and improving motor performance (Strand et al., 2014). Disparities between strength on distal (e.g., hand) and proximal musculature (e.g., upper limb and trunk muscles) in musicians have been reported previously (Ackermann et al., 2002; Driscoll and Ackermann, 2012). In addition, musicians must often adopt awkward positions when playing their instruments, requiring flexibility and strength that, if lacking, may expose them to risk of injury (Heming, 2004). Our results indicate that bespoke exercise programs for musicians that focus on upper body strength may be relevant, also paving the way for future research to scrutinize their impact on injury prevention and treatment, as well as performance. In a previous study by Chan et al. (2013a, 2014), exercises focusing on scapular and rotator cuff stability were considered appropriate for inclusion in a musician-centered program in restoring shoulder muscle balance and movement control, as well as other exercises focusing on improving abdominal and hip strength. Andersen et al. (2017) also highlight the potential of strength and general fitness training for increasing musicians’ motivation and positive attitudes toward exercise, as well as reducing pain and increasing aerobic capacity. Existing studies (Kava et al., 2010; Wasley et al., 2012; Chan et al., 2013b, 2014; Andersen et al., 2017) point to the need for exercise training to improve muscular endurance, postural control and strength, as well as to reduce pain. In fact, the positive effects of exercise for both physical and psychological health among other populations are widely documented (Broman-Fulks et al., 2004; Nawrocka et al., 2013), yet there appears to be a lack of specific exercise training and education available for musicians in educational and professional settings.

While our findings suggest that music students are engaging in weekly physical activity with cardiovascular benefits, it appears that evidence of regular engagement in muscle-strengthening activities is still lacking. Unfortunately, many music students may believe that exercising is risky, especially muscle-strengthening activities, and that it can cause muscle fatigue which may then have a negative impact on practice and performance (Chan et al., 2013b). Despite widely published recommendations on the importance of exercise and physical activity for health generally, specific evidence in music is limited and only one study has examined the impact of strengthening and endurance training for music students. Ackermann et al. (2002) demonstrated improvements in muscle strength and a perceived reduction of symptoms of performance-related musculoskeletal disorders and exertion while playing. While the changes observed were small, the study showed the relevance of both strength and endurance training for musicians, and students perceived them as important to their musical pursuits. Nonetheless, the perceived importance of exercise and motives for physical activity for music students are still largely unknown and should be investigated further to shed light on possible barriers to behavioral change, as well as to inform the design of relevant and motivating exercise interventions.

In our study, some differences were observed between instrument groups and levels of study, suggesting that the physical and physiological demands of music making may be instrument- and training-specific; therefore, exercise recommendations should fit the specific needs of instrument groups at different career stages. Whether these differences result from instrument selection practices, individual differences, and/or from the impact of years of practice leading to anatomical and physiological changes remains to be seen (Driscoll and Ackermann, 2012). Observed differences between levels of study indicate that instrument specialization, which also reflects cumulative years of practice, may have an impact on musicians’ health-related fitness. However, caution is needed when interpreting these findings. Small effect sizes suggest that these differences may not be relevant in practice. In addition, inevitable uneven sample distributions and the potential mediating effect of sex, with different distributions of women and men across instrument groups, may have affected the results. Finally, this was a self-selected sample with a great majority of participants volunteering from elite training institutions mostly in Western classical music, and our results may predominantly represent those music students who are already aware of and committed to enhancing their health-related fitness. It would be instructive for future studies to reach a wider representation of music students, as well as explore comparisons between those musicians at different stages of their education and career in order to understand better the potential effects of practice and training on musicians’ fitness and the fitness requirements to meet the demands of music making.

## Conclusion

Physical fitness should be taken seriously in music education settings and considered an integral part of comprehensive musical training, informed by the demands of the profession. By deliberately including learning and support services related to health and wellbeing—physical, as well as psychological and emotional—in students’ timetables and by expanding health-related provision more generally, we can increase knowledge, active participation, and responsibility for health matters across the sector.

Firstly, we suggest that fitness monitoring in conservatoires and specialist music schools is needed to inform educational practices and raise awareness. This could translate into health-related and functional fitness assessments that identify areas to be targeted for injury prevention and health enhancement. Secondly, we argue that music students should be supported in learning about the structure and function of the body, particularly in relation to the specialisms in which they perform (e.g., instruments and genres); this could help clarify for them the relevance of looking after their bodies properly both for general health literacy and for meeting music-specific demands. Finally, our findings suggest that, while music students’ current levels of fitness are generally satisfactory within the wider picture of university-level students, enhancement of upper body strength and endurance could be beneficial. Indeed, we would urge the development of strength and conditioning training, tailored to performance specialisms, both within curricula and as supplemental activities. Increasing upper body strength will help musicians face the physical stresses of practicing, repetitive movements, and carrying and holding heavy instruments, often in asymmetrical body positions.

Overall, redesigning specialist music training with whole-system, context-driven, and comprehensive approaches is required so that music students are better prepared to face the changing landscape and the multiple demands of the music profession. We acknowledge the limited resources available in most conservatoires, and so, education through regular workshops and seminars, sessions with health and exercise professionals who deliver music-specific fitness routines, partnerships with gyms and fitness studios for health screenings and affordable access to fitness facilities, and exercise challenges promoted by staff and students are all creative ways of engaging musicians in promoting their health and wellbeing.

## Data Availability Statement

The datasets generated for this study are available on request to the corresponding author.

## Ethics Statement

The studies involving human participants were reviewed and approved by the Conservatoires UK Research Ethics Committee. The participants provided their written informed consent to participate in this study.

## Author Contributions

All authors listed have made a substantial, direct and intellectual contribution to the work, and approved it for publication.

## Conflict of Interest

The authors declare that the research was conducted in the absence of any commercial or financial relationships that could be construed as a potential conflict of interest.

## Footnotes

1 http://hypermobility.org

2 http://cooperinstitute.org/fitnessgram

3 https://sites.google.com/site/theipaq/
