# Supplementary material for: Fit to Perform: A Profile of Higher Education Music Students’ Physical Fitness
Source: Front Psychol. 2020 Mar 5;11:298. doi: 10.3389/fpsyg.2020.00298 (PMC7066496; doi:10.3389/fpsyg.2020.00298)
Supplement: Supplementary file 1 [file Data_Sheet_1.pdf]

Araújo LS, Wasley D, Redding E, Atkins L, Perkins R, Ginsborg J and Williamon A (2020), Fit to Perform: A Profile of Higher Education Music Students' Physical Fitness, *Front. Psychol.* 11:298. doi: 10.3389/fpsyg.2020.00298.

SUPPLEMENTARY TABLE 1 | Descriptive statistics for the health-related fitness indicators used in the *Fit to Perform* screening protocol by instrument group and sex.

**Measure**	**Strings**		**Keyboard**		**Woodwinds**		**Brass**		**Voice**		**Percussion**		**Other**	
	**Women**	**Men**	**Women**	**Men**	**Women**	**Men**	**Women**	**Men**	**Women**	**Men**	**Women**	**Men**	**Women**	**Men**
**Lung function**	**M ± SD**													
FEV_1	2.80 ±0.63	3.86 ±0.95	2.67 ±0.63	3.64 ±1.05	3.03 ±0.42	3.74 ±0.98	3.15 ±0.52	4.70 ±0.61	2.92 ±0.49	3.52 ±0.38	-	3.72 ±0.95	3.01[1]	4.42 ±0.86
FEV_1 %pred	86.83 ±17.69	88.46 ±20.41	87.61 ±19.94	87.43 ±21.96	91.91 ±11.27	85.18 ±19.54	97.75 ±13.75	102.69 ±9.80	89.44 ±14.12	86.14 ±7.99	-	82.00 ±22.07	95.00[1]	99.75 ±12.63
FVC	2.95 ±.67	4.16 ±1.07	2.81 ±0.69	3.95 ±1.07	3.30 ±0.50	5.16 ±1.95	3.44 ±.68	5.17 ±0.85	3.09 ±0.57	3.81 ±0.63	-	4.34 ±0.80	3.01[1]	4.77 ±0.87
FVC %pred	79.10 ±16.69	80.42 ±19.39	80.22 ±18.59	80.64 ±18.89	87.42 ±11.73	99.45 ±34.30	93.13 ±15.09	93.31 ±13.57	82.56 ±14.87	78.86 ±11.63	-	80.00 ±16.64	82.00[1]	91.00 ±10.95
FEV_1/FVC %	97.24 ±3.88	94.96 ±5.97	98.11 ±1.94	94.36 ±7.12	95.03 ±5.85	86.18 ±12.48	92.87 ±6.22	93.23 ±7.89	96.60 ±3.98	95.86 ±5.08	-	87.67 ±12.06	100.00[1]	92.75 ±6.18
FEV_1/FVC %pred	115.50 ±4.81	114.92 ±7.24	116.17 ±2.38	114.07 ±8.80	112.67 ±7.00	104.18 ±15.22	110.38 ±7.41	112.77 ±9.51	114.52 ±4.89	116.57 ±6.70	-	106.33 ±14.57	122.00[1]	112.25 ±7.46

cont…/

Measure	Strings		Keyboard		Woodwinds		Brass		Voice		Percussion		Other	
	Women	Men	Women	Men	Women	Men	Women	Men	Women	Men	Women	Men	Women	Men
**Flexibility and range of motion**							**% (n)**							
AT 1_R	93% (39)	100% (26)	94% (17)	93% (13)	97% (32)	91% (10)	100% (8)	100% (13)	100% (25)	100% (7)	-	100% (3)	100% (1)	100% (4)
AT 1_R pain	7% (3)	0% (0)	0% (0)	7% (1)	0% (0)	27% (3)	0% (0)	8% (1)	4% (1)	0% (0)	-	0% (0)	100% (1)	0% (0)
AT 1_L	93% (39)	96% (25)	100% (18)	93% (13)	97% (32)	73% (8)	100% (8)	100% (13)	100% (25)	100% (7)	-	100% (3)	100% (1)	100% (4)
AT 1_L pain	5% (2)	4% (1)	0% (0)	7% (1)	0% (0)	9% (1)	0% (0)	0% (0)	0% (0)	14% (1)	-	0% (0)	0% (0)	0% (0)
AT 2_R	81% (34)	62% (16)	89% (16)	57% (8)	79% (26)	82% (9)	100% (8)	92% (12)	76% (19)	71% (5)	-	100% (3)	0% (0)	100% (4)
AT 2_R pain	12% (5)	19% (5)	11% (2)	29% (4)	21% (7)	18% (2)	0% (0)	23% (3)	16% (4)	14% (1)	-	0% (0)	100% (1)	0% (0)
AT 2_L	91% (38)	81% (21)	89% (16)	86% (12)	88% (29)	91% (10)	100% (8)	100% (13)	96% (24)	100% (7)	-	100% (3)	100% (1)	75% (3)
AT 2_L pain	5% (2)	12% (3)	6% (1)	7% (1)	6% (2)	9% (1)	0% (0)	0% (0)	4% (1)	14% (1)	-	0% (0)	0% (0)	25% (1)
							**M ± SD**							
Beighton	2.07 ±1.42	1.54 ±1.56	2.50 ±2.18	.93 ±1.14	1.97 ±2.31	2.09 ±1.70	2.75 ±1.83	1.31 ±1.38	2.52 ±2.12	1.14 ±1.21	-	.33 ±.58	0[1]	4.75 ±2.99
R stretch	3.64 ±.91	3.35 ±1.38	3.61 ±.98	3.36 ±1.15	3.45 ±.94	3.55 ±1.21	3.50 ±1.07	3.92 ±.28	3.76 ±.66	4.00 ±.0	-	4.00[1]	4.00[1]	2.75 ±1.89
L stretch	3.21 ±1.32	2.69 ±1.54	3.06 ±1.47	2.00 ±1.75	2.45 ±1.48	3.09 ±1.45	3.13 ±1.13	3.69 ±.75	3.24 ±1.16	3.14 ±1.21	-	4.00[1]	0[1]	2.75 ±.96
Sit and reach	28.71 ±9.51	24.27 ±11.65	30.19 ±12.04	18.21 ±8.88	27.52 ±10.74	24.82 ±10.18	29.75 ±10.86	21.92 ±13.60	32.72 ±10.08	32.29 ±12.42	-	25.33 ±8.74	22.00[1]	30.25 ±11.87

cont…/

Measure	Strings		Keyboard		Woodwinds		Brass		Voice		Percussion		Other	
	Women	Men	Women	Men	Women	Men	Women	Men	Women	Men	Women	Men	Women	Men
**Strength and endurance**							**M ± SD**							
HG-R	26.37 ±5.12	39.62 ±7.17	26.20 ±3.95	36.74 ±6.70	27.54 ±5.20	39.81 ±9.24	26.34 ±5.18	43.19 ±6.22	27.07 ±4.63	36.01 ±6.25	25.59 ±2.62	42.69 ±8.73	27.05 ±2.86	39.03 ±6.55
HG-L	25.41 ±5.09	37.65 ±6.59	24.70 ±3.94	34.96 ±5.54	26.05 ±4.80	38.23 ±8.53	25.37 ±3.39	40.53 ±4.28	25.89 ±3.85	35.61 ±4.95	24.17 ±2.34	40.11 ±8.82	26.75 ±1.52	37.83 ±6.53
Plank	49.57 ±15.66	52.92 ±18.04	49.22 ±14.69	59.50 ±1.87	51.64 ±12.17	55.27 ±9.63	52.13 ±13.48	54.38 ±14.04	56.96 ±11.04	54.57 ±14.36	-	60.00[1]	60.00[1]	55.25 ±6.18
Press-up	12.40 ±9.84	22.00 ±16.12	8.99 ±7.93	19.57 ±12.06	9.18 ±7.03	25.64 ±15.13	7.75 ±4.17	17.23 ±7.55	13.40 ±8.36	19.29 ±9.14	-	14.33 ±10.02	0[1]	17.25 ±18.68
**Cardiovascular fitness**							**M ± SD**							
RecHR	106.68 ±16.74	100.50 ±17.83	108.14 ±14.70	95.38 ±15.39	102.36 ±19.37	98.30 ±13.03	109.17 ±15.90	100.82 ±18.24	105.29 ±17.04	104.64 ±24.64	99.00 ±3.85	90.25 ±25.23	94.00 ±12.77	105.29 ±18.17
**Physical activity**							**M ± SD**							
Walking	1285.82 ±1218.24	1093.44 ±906.52	954.25 ±800.21	795.54 ±746.57	1665.00 ±1352.83	583.50 ±483.78	1270.50 ±1033.06	865.62 ±490.38	1556.28 ±1045.56	1560.43 ±819.42	-	2194.50 ±1709.24	330.00[1]	841.50 ±365.99
Moderate	220.48 ±370.40	501.54 ±758.46	328.89 ±501.91	445.71 ±735.87	680.00 ±866.33	349.09 ±357.81	295.00 ±362.18	501.54 ±921.10	941.60 ±1272.14	900.00 ±608.93	-	560.00 ±969.95	480.00[1]	420.00 ±494.77
Vigorous	618.10 ±1007.61	1024.62 ±1773.48	148.89 ±245.35	1057.14 ±1928.23	722.42 ±1346.92	1200.00 ±989.55	150.00 ±285.06	812.31 ±938.00	905.60 ±1114.44	2537.14 ±3413.45	-	853.33 ±1211.50	480.00[1]	660.00 ±1025.28
Total PA	2124.39 ±1777.47	2619.60 ±2226.43	1432.03 ±1014.06	2298.39 ±2489.30	3067.42 ±2017.40	2132.59 ±1446.23	1715.50 ±1015.38	2179.46 ±1089.15	3403.48 ±2557.00	4997.57 ±3829.18	-	3607.83 ±3871.30	1290.00[1]	1921.50 ±1292.56

Note. M=mean; SD=standard deviation. Measures, abbreviations, and units for each measure are provided in Table 2. *Strings:* violin, viola, viola de Gamba, cello, double bass, guitar (classical and electric), and harp; *Keyboard:* accordion, piano, organ, harpsichord, and historical keyboards; *Woodwinds:* flute, recorder, clarinet, oboe, bassoon, and saxophone; *Brass:* cornet, euphonium, horn, trombone, trumpet, and tuba; *Other:* composition and conducting. [1] Values arise from small n values (n<3).
