# Supplementary material for: Fit to Perform: A Profile of Higher Education Music Students’ Physical Fitness
Source: Front Psychol. 2020 Mar 5;11:298. doi: 10.3389/fpsyg.2020.00298 (PMC7066496; doi:10.3389/fpsyg.2020.00298)
Supplement: Supplementary file 2 [file Data_Sheet_2.pdf]

Araújo LS, Wasley D, Redding E, Atkins L, Perkins R, Ginsborg J and Williamon A (2020), Fit to Perform: A Profile of Higher Education Music Students' Physical Fitness, *Front. Psychol.* 11:298. doi: 10.3389/fpsyg.2020.00298.

**SUPPLEMENTARY TABLE 2 |** Descriptive statistics for the health-related fitness indicators used in the *Fit to Perform* screening protocol by level of study, as well as Mann Whitney U tests for differences by sex.

| Measure | Undergraduate | | | Postgraduate | | |
|---|---|---|---|---|---|---|
| | Women | Men | U, p | Women | Men | U, p |
| **Lung function** | M ± SD | | | M ± SD | | |
| $FEV_1$ | 2.93±0.58 | 4.18±0.75 | **241.00, p<.001** | 2.84±0.54 | 3.62±1.06 | **529.50, p<.001** |
| $FEV_1$ %pred | 90.28±14.91 | 94.73±13.38 | 1201.00, p=.157 | 88.74±16.48 | 84.59±22.70 | 949.50, p=.423 |
| FVC | 3.12±0.65 | 4.50±0.88 | **261.50, p<.001** | 3.04±0.61 | 4.36±1.59 | **443.50, p<.001** |
| FVC %pred | 83.34±14.87 | 85.43±12.92 | 1364.00, p=.683 | 82.66±16.54 | 86.00±28.60 | 975.50, p=.547 |
| $FEV_1$/FVC % | 96.62±4.55 | 95.00 ±6.52 | 1399.50, p=.847 | 96.19±4.72 | 90.44±9.49 | **638.50, p=.001** |
| $FEV_1$/FVC %pred | 114.45±5.43 | 114.93 ±7.88 | 1076.50, p=.280 | 114.34±5.83 | 109.53±11.72 | 836.00, p=.093 |
| **Flexibility and range of motion** | % (n) | | | % (n) | | |
| AT 1_R | 99% (64) | 98% (43) | 1419.50, p=.780 | 94% (58) | 97% (33) | 1017.00, p=.461 |
| AT 1_R pain | 3% (2) | 5% (2) | 1409.00, p=.780 | 5% (3) | 9% (3) | 1010.00, p=.443 |
| AT 1_L | 100% (65) | 93% (41) | **1332.50, p=.034** | 94% (58) | 94% (32) | 1048.00, p=.913 |
| AT 1_L pain | 2% (1) | 5% (2) | 1387.00, p=.349 | 2% (1) | 6% (2) | 109.00, p=.253 |
| AT 2_R | 85% (55) | 71% (31) | 1227.50, p=.077 | 77% (48) | 77% (26) | 1044.00, p=.916 |
| AT 2_R pain | 12% (8) | 16% (7) | 1378.50, p=.594 | 18% (11) | 24% (8) | 993.00, p=.448 |
| AT 2_L | 92% (60) | 91% (40) | 1410.00, p=.796 | 90% (56) | 85% (29) | 1001.00, p=.462 |
| AT 2_L pain | 5% (3) | 9% (4) | 1366.00, p=352 | 5% (3) | 9% (3) | 1012.00, p=.443 |
| | M ± SD | | | M ± SD | | |
| Beighton | 1.75±1.98 | 0.86±1.17 | **1093.00, p=.028** | 2.71±1.81 | 2.44±1.86 | 947.00, p=.404 |
| R stretch | 3.55±1.04 | 3.48±1.23 | 1419.50, p=.925 | 3.66±.67 | 3.59±1.02 | 1001.00, p=.591 |
| L stretch | 2.94±1.53 | 2.88±1.56 | 1418.50, p=.935 | 3.00±1.21 | 2.88±1.34 | 1012.50, p=.733 |
| Sit and reach | 29.38±10.09 | 21.59±11.53 | **862.00, p<.001** | 29.45±10.79 | 26.67±11.13 | 917.50, p=.295 |

*cont…/*

| Measure | Undergraduate | | | Postgraduate | | |
|---|---|---|---|---|---|---|
| | Women | Men | U, p | Women | Men | U, p |
| **Strength and endurance** | **M ± SD** | | | **M ± SD** | | |
| HG-R | 26.53±4.91 | 38.85±6.91 | 1723.00, p=.935 | 26.95±4.68 | 40.91±8.76 | **474.50, p<.001** |
| HG-L | 25.28±4.64 | 37.08±6.32 | **1723.00, p<.001** | 25.79±4.39 | 38.83±7.18 | **371.00, p<.001** |
| Plank | 51.29±12.05 | 51.66±16.70 | 1252.00, p=.218 | 52.24±15.37 | 59.82±1.03 | 995.00, p=.646 |
| Press-up | 10.89±8.94 | 18.32±12.19 | **853.00, p<.001** | 10.85±8.02 | 23.32±14.47 | **472.00, p<.001** |
| **Cardiovascular fitness** | **M ± SD** | | | **M ± SD** | | |
| RecHR | 104.55±17.04 | 98.14±17.45 | **10149.50, p=.001** | 107.17±16.69 | 102.42±18.01 | 2310.50, p=.090 |
| **Physical activity** | **M ± SD** | | | **M ± SD** | | |
| Walking | 1338.53±1067.80 | 1135.13±883.98 | 1292.00, p=.392 | 1427.78±1274.41 | 828.40±726.53 | **797.50, p=.049** |
| Moderate | 379.38±515.78 | 563.18±730.87 | 1243.00, p=.233 | 634.52±1029.72 | 427.06±692.94 | 929.50, p=.328 |
| Vigorous | 665.23±1173.66 | 1185.45±1701.98 | **110.50, p=.039** | 541.29±910.52 | 1058.82±1867.99 | 847.50, p=.097 |
| Total PA | 2383.15±1597.74 | 2883.76±2264.87 | 1215.00, p=.478 | 2603.59±2362.04 | 2314.28±2379.27 | 986.00, p=.602 |

*Note.* M=mean; SD=standard deviation. Measures, abbreviations, and units for each measure are provided in Table 2.
